# Supplementary material for: A qualitative study on the challenges of Afghan child labourers in Tehran
Source: PLoS One. 2024 Jul 12;19(7):e0306318. doi: 10.1371/journal.pone.0306318 (PMC11244830; doi:10.1371/journal.pone.0306318)
Supplement: S1 File — (DOCX) [file pone.0306318.s001.docx]

**Interview Guide**

Before constructing this interview guide, we explored literature on child labourers and chose three important life areas to focus the interviews on, namely: Psychological challengesو Health-related challenges and Social challenges.

This interview guide was developed in cooperation with healthcare professionals (i.e.Sociology, psychology and health education, and health promotion) who are experts in the areas we were interested in. The interview guide consists of an introduction and open-ended questions about sociodemographic and information, and the six areas of interest: Psychological challenges, Health-related challenges and Social challenges. More specifically, the guide consists of these questions:

1. How do you feel about working in Iran as a child manual worker?
2. What sorts of problems have you experienced so far while working here?
3. How do people react to you working as an Afghan child here?
4. How do your employer and co-workers treat you in your workplace?
5. What do you think about the government and the social security organisations in Iran?
6. What are the main challenges for Afghan child labourers in Iran?

**Introduction**

Thank you for being willing to share your opinions and experiences about your child labourer with me. Before we start, I want to assure you that your interview answers will be kept confidential. Your feedback will be combined with that of other study participants and will not be linked to your name. You may skip any questions you don’t care to answer and you may end the interview at any time.

We would like to record this interview to make sure we listed your answers correctly. Your name will not be associated with the recording or what has been transcribed; a study ID # will be assigned instead. We will destroy the recording when we are finished with the study. In order to help protect your privacy, please do not to state your name while the recorder is on. If your name or other information that identifies you does come up in the written transcripts, we will delete it before sharing with the study researchers.
