# Supplementary material for: A qualitative study on the challenges of Afghan child labourers in Tehran
Source: PLoS One. 2024 Jul 12;19(7):e0306318. doi: 10.1371/journal.pone.0306318 (PMC11244830; doi:10.1371/journal.pone.0306318)
Supplement: S1 Table — Each quote within each theme is from a different study participant. (DOCX) [file pone.0306318.s002.docx]

**Table S1: Complete list of study themes and representative quotes. Each quote within each theme is from a different study participant.**

| **Example Quotes** | Codes | Sub-categories | Categories |
| --- | --- | --- | --- |
| *"There is no single day without a fight with them. They keep swearing at me or even beating me." (10-year-old boy)*  *"Many times, as soon as the drivers find out we are Afghans, they insult us and begin to swear." (11-year-old boy)*  *"Some drivers hit us on purpose or make us work and tell us to clean the window, but as soon as we clean, they fail to pay and just drive away." (13-year-old boy)*  *"It happened several times that the municipal officials came and took our belongings with them or broke everything on the spot, but there was nothing we could do about it." (9-year-old girl)*  *Once, when the street was quiet, someone in a car called me and said, Come here; I’d like to buy chewing gum from you. As soon as I approached, he grabbed my hand and forced his hand under my clothes and (12-year-old girl)*  *"So far, it has happened many times that I worked till night and collected money, but bullies came at night and took my money away and even beat me." (14-year-old boy)* | Experiencing verbal harassment, mental harassment, physical and sexual abuse in the street, being abused as an Afghan, being beaten by municipal officials, blackmailing informal foremen, being robbed of income | History of harassment or abuse | Psychological challenges |
| *"When I see other children of my age who are in billion-dollar cars and everything is provided for them, I feel sad and jealous of them." (8-year-old boy)*  *"Sometimes I like to scratch and damage the expensive cars I wash." (7-year-old boy)*  *"I am a girl. I am always afraid that someone hurts me or arrests me and sends me back to Afghanistan." (9-year-old girl)*  *"I got so annoyed that I don't trust anyone anymore; even sometimes, when someone wants to be kind to me and give me some food or something, I'm afraid to approach him because I tell myself he may hurt me." (11-year-old girl)* | Jealousy towards peers, revenge and hostility towards the rich, instability and restlessness, mistrust of others, anxiety, a sense of insecurity and fear, fear of deport to Afghanistan | Negative effects |  |
| *"I have a row at least once or twice a day. Is it possible to work at a crossroads and not fight? Sometimes I fight with a driver, sometimes with other people." (14-year-old boy)*  *"Sometimes I lift a phone when I get a chance." (8-year-old girl)*  *"I often get tired of life. I was going to commit suicide once or twice. I cut a vein in my hand once, but I didn't die." (13-year-old boy)*  *"It happened several times when I was offered some money and asked to deliver a package of drugs. I did so, though I knew the hazards. I only agreed just because they paid a lot of money." (8-year-old girl)*  *"I have been smoking cigarettes and hash for years." (14-year-old boy)*  *"Several times, I had sex with a neighbour's girl, who is also Afghan." (13-year-old boy)* | aggressiveness, theft, suicide, drug abuse or transport, and high-risk sexual behaviours | High-risk behaviours |  |
| *"I don't have strong feelings for my parents because I never see them properly. Every morning, I leave home and am in the streets until night, when I go back home. My father is a doorman, and we do not see him at all." (10-year-old girl)*  *"When I think that my parents gave birth to me in these terrible conditions, I don't feel good about them. I feel they were selfish for giving birth to me, so I can't really love them." (13-year-old girl)*  *"I don't need my parents; I make money myself. Often I don't tell them where I work and what I do." (11-year-old boy)*  *"Sometimes I don't go home for two weeks, and I stay with a friend, and I don't tell anyone where I am." (14-year-old boy)*  *"My father wants to give him all the money I earn, and sometimes I hide it from him. We have argued several times over this issue." (9-year-old boy)* | Less time at home, no strong emotional bond with parents, economic independence from the family, lack of parental supervision, perceived redundancy of parents, tension and conflict with parents | Family detachment |  |
| injuries in them that can threaten their health.  *"Sometimes I get so tired that I faint." (5-year-old girl)*  *"Working at the crossroads is so hard in winter because it gets very cold and you can't light a fire to warm yourself." (7-year-old boy)*  *"When I go home at night, I don't know how to sleep; my whole body hurts; I feel like I am dead on my feet." (15-year-old boy)*  *"Sometimes I do not take off my shoes for 12 hours. When I do, I see my feet are blistered and sore." (14-year-old boy)* | Physical weakness, sunburn, cold, back pain, sore toes, pain in hands and feet, digestive problems, skin problems | Physical problems | Health-related challenges |
| *"Most of our neighbours are drug dealers." (8-year-old girl)*  *"Our neighbourhood is very poor. There are many fights during the day. There are a lot of thefts. Overall, when we reach the street where we live, we feel terrified and insecure, and no one can stop us." (15-year-old boy)*  *"We live with the family of three of my uncles in an old 60-metre house." (11-year-old girl)*  *"Too often I can't sleep properly at night. There are ten of us in a small room." (12-year-old boy)*  *"It's our turn to take a bath. Sometimes we can't take a bath for several weeks." (9-year-old girl)* | Living in poor and high-risk areas (buying and selling drugs nearby), inadequate number of rooms, housing large populations in small and unsanitary areas, several families living in one small area, failure to maintain hygiene at home | Inappropriate accommodation |  |
| *"When we get sick, if we feel terrible, we visit a doctor because our expenses are very high and we don't have any insurance." (8-year-old boy)*  *"Some doctors and nurses do not behave properly when they find out that I am an Afghan. Sometimes they insult us." (11-year-old boy)*  *"I was born in Iran because we came there illegally. Fearing that they would pick on us, I didn't go for any vaccinations, even those that were free." (12-year-old girl)* | No health insurance, high cost of medication in Iran, the health staff’s inappropriate behaviours towards Afghans, no vaccination | Medical and therapeutic problems |  |
| *"Since the beginning of the COVID-19 pandemic, because I am always out there in the streets, I have been infected several times. It bothered me a lot because I could not even visit a doctor." (9-year-old boy)*  *"One or two of my friends got hepatitis; I think maybe because they cut their hands while scavenging several times with broken glass." (15-year-old boy)*  *"I eat out most days; I mostly eat cold food." (6-year-old girl)*  *"The days when the air is polluted, I find it really hard. There have been several times that I fainted." (9-year-old girl)*  *"So far, I have had several accidents and been hit by a car; once I broke a leg and had to come to work with a broken leg." (6-year-old boy)* | Exposure to infection (AIDS, hepatitis, COVID-19, etc.), improper nutrition, exposure to adverse weather conditions, car accidents, exposure to accidents | Health threat |  |
| *"When I'm at home and I'm unemployed, I watch more TV because I don't have any toys to entertain myself with; sometimes I feel like I'm not a kid at all." (7-year-old boy)*  *"All my time is spent in the streets; that's why I don't get any chance to play or go to the park. I don't remember the last time I went to the park and played. I want to be a child and play and be like other children." (11-year-old boy)*  *"I was 12 years old when I married a man who was much older than me. I lived with him for a year, and then he returned me to my family." (15-year-old girl)* | No fun, toy, or amusement; no spare time to spend with friends; no exercise; no time to play; early marriage (for girls) | Neglected childhood | Social challenges |
| *"I was born in Iran. I have never been to Afghanistan. I really don't know if I should call myself Iranian or Afghan." (11-year-old girl)*  *"I don't even know how old I am; I don't have any birth certificate or document to show how old I am." (9-year-old girl)*  *"I was born in Iran and grew up here. Even my father was born in Iran, but no one recognises us as Iranians." (15-year-old boy)*  *"Not knowing how old you are and when your birthday is really bothers me." (8-year-old boy)* | No enthusiasm for and interest in the homeland (Afghanistan), failure to be accepted in Iran, lack of identity certificates, sense of alienation | Dual identity |  |
| *"My family and I are here illegally; that's why I can't go to school; I don't even know how to read and write." (13-year-old girl)*  *"We live in an area where there is no school that enrols us Afghans; that's why my father didn't let me go to school because if I did, I would have to move away from home." (8-year-old girl)*  *"My father and I went to enrol at a school. The school principal insulted us and said, This is not the place for you Afghans." (10-year-old boy)*  *"I went to school for a couple of years, but the teachers and administrators treated me so badly that I stopped going there." (11-year-old boy)*  *"Many children insulted me in class and even beat me. I didn't care about school anymore." (13-year-old boy)* | Restrictions on enrollment in schools due to illegal attendance, few schools for Afghan children, refusal of some schools to enrol Afghan children, negative attitude of school administrators and students towards Afghan children | Educational problems |  |
| *"If something happens to me, I have to deal with it myself. There is nowhere to go to defend my rights." (14-year-old boy)*  *"Sometimes the municipality interferes and organises a class for us that tells us to behave in a way that we will be less injured, but these classes are very few in number, and most of the time no one attends them." (11-year-old girl)*  *"Until now, no workshop or class has been organised for me to tell me how to protect myself in the streets." (12-year-old girl)*  *"I have been beaten many times, but there is no place to go to complain. If I go to the police, they will arrest me because I am an illegal resident in Iran." (13-year-old boy)* | Lack of supportive social organisations, lack of coherent programmes to improve children's health, lack of appropriate educational plans for child labourers | Inadequate social support |  |
| *"I have almost no friends; I do not get close to anyone. I often like to have a relationship with others, but as soon as they see I am Afghan, hardly anyone is willing to make friends with me." (8-year-old girl)*  *"I made friends with an Iranian boy at school. One day he invited me to his house, but his parents treated me very badly as soon as they saw that I was Afghan, and I heard them telling their son that he could not invite me home." (12-year-old boy).*  *"Our family only socialises with our relatives in Iran, who are also in other cities and rarely visit us." (6-year-old girl)*  *"When I'm not working, I'm always at home; I don't have anywhere to go. We don't go to parties at anyone's house, and no one comes to visit us either." (10-year-old boy)*  *"If I want to make friends with someone, I have to change my clothes and go to another place to sell things because of the municipal officials; that's why I don't have any close friends." (14-year-old boy)* | Lack of social relations, isolation (no contact with Iranian families), loneliness, and not enough friends due to the change of work position and inability to communicate with Iranian children | Social isolation |  |
| *"Because of my appearance, it is obvious that I am Afghan; that's why they keep insulting me very often." (14-year-old boy)*  *"I was working somewhere, and as soon as they found out I am Afghan, they began to insult me and call me by different insulting names before I stopped working there anymore." (13-year-old boy).*  *"Sometimes when I'm in the streets, I get insulted a lot. They use very abusive words to call me or my family. I can't do anything about it." (10-year-old boy)* | Humiliation in the street, humiliation at work, inappropriate nicknames because of being an refugees, being socially labelled | Social humiliation |  |
