# Supplementary material for: A qualitative study on the challenges of Afghan child labourers in Tehran
Source: PLoS One. 2024 Jul 12;19(7):e0306318. doi: 10.1371/journal.pone.0306318 (PMC11244830; doi:10.1371/journal.pone.0306318)
Supplement: Attachment — Submitted filename: Response to Reviewers.docx [file pone.0306318.s004.docx]

**Dear Editor and Reviewers,**

We appreciate the interest that the editor and reviewers have taken in our manuscript and the constructive comments they have given. We have addressed the concerns of the reviewers and provided a point-by-point response to the comments. Additionally, we have highlighted the modifications in the paper.

Thank you again for consideration of our revised manuscript.

Sincerely,

Reviewers' comments:

Reviewer's Responses to Questions

**Comments to the Author**

1. If the authors have adequately addressed your comments raised in a previous round of review and you feel that this manuscript is now acceptable for publication, you may indicate that here to bypass the “Comments to the Author” section, enter your conflict of interest statement in the “Confidential to Editor” section, and submit your "Accept" recommendation.

Reviewer #2: All comments have been addressed

Reviewer #3: All comments have been addressed

Reviewer #2: (No Response)

Reviewer #3: The study is an interesting one and the authors' made efforts at following standard academic research procedures to investigate the issue under discourse. I carefully read through the manuscript and I found it academically stimulating and contributory to the discourse and knowledge of child welfare policy and protection.
The authors followed the comments of the earlier reviewers, and implemented same in the manuscript which helped to improve the quality of the manuscript. Regarding making all data underlying the findings in the manuscript fully available, the authors explained that "The data are not publicly available due to their containing information that could compromise the privacy of research participants as the data contain potentially identifying child labourers information". However, the authors further stated that "Interview guide and Complete list of study themes and representative quotes is included as supplementary material. It is required that all researchers who wanted to work on this data submit their data request access". The position of the authors are ethical and should be respected.
Whereas I consider the manuscript standard enough for publication in its current form, there are two key observations I made. 1. Page 1 line 1 under abstract, the last in the sentence should be "refugees" in line with the subject matter (Afghan child labourers...).
2. Page 5 line 6 under ethical considerations, the sentence that started with "also", should be in initial Cap (Also,...). The manuscript has therefore improved to meet the journal's standard for publication after the minor corrections.

Response:we have highlighted the modifications in the paper.

Thank you again for consideration of our revised manuscript.

Sincerely,
